# Effectiveness of Trainings of General Practitioners on Antibiotic Stewardship: Methods of a Pragmatic Quasi-Experimental Study in a Controlled Before-After Design in South-East-Lower Saxony, Germany (WASA)

**DOI:** 10.3389/fphar.2021.533248

**Published:** 2021-04-22

**Authors:** Daniela Gornyk, Martina Scharlach, Brigitte Buhr-Riehm, Carolina Judith Klett-Tammen, Sveja Eberhard, Jona Theodor Stahmeyer, Anika Großhennig, Andrea Smith, Sarah Meinicke, Wilfried Bautsch, Gérard Krause, Stefanie Castell

**Affiliations:** ^1^ Department of Epidemiology, Helmholtz Centre for Infection Research, Braunschweig, Germany; ^2^ PhD Programme Epidemiology Hannover-Braunschweig, Braunschweig, Germany; ^3^ Governmental Institute of Public Health of Lower Saxony, Hannover, Germany; ^4^ Fachbereich Soziales und Gesundheit, Braunschweig, Germany; ^5^ Centre for Public Health and Healthcare, Hannover Medical School, Hannover, Germany; ^6^ AOK Niedersachsen-Statutory Health Insurance of Lower Saxony, Hannover, Germany; ^7^ Institut für Biometrie, Hannover Medical School, Hannover, Germany; ^8^ Technische Universität Braunschweig, Braunschweig, Germany; ^9^ Institute of Microbiology, Immunology and Hospital Hygiene, City Hospital Brunswick, Braunschweig, Germany; ^10^ Hannover Medical School, Hannover, Germany

**Keywords:** primary health care, prescribing behaviour, antibiotic resistance, general practitioner, antibiotic stewardship, primary and secondary data

## Abstract

**Introduction:** Antibiotic resistance is a serious threat to global public health. It reduces the effectiveness of treatments for serious bacterial infections and thus increases the risk of fatal outcomes. Antibiotic prescriptions are often not in line with clinical evidence-based guidelines. The process of emergence of resistant bacteria can be slowed down by adherence to guidelines. Yet this adherence seems to be lacking in primary health care.

**Methods and Analysis:** This pragmatic quasi-experimental study using a controlled before-after design was carried out in South-East-Lower Saxony in 2018–2020. The voluntary attendance of interactive trainings with condensed presentation of current guidelines for general practitioners (GP) on antibiotic management for urinary and respiratory tract infections is regarded as intervention. Those GP not attending the trainings constitute the control group. Data were collected via questionnaires; routine health records are provided by a statutory health insurance. The primary outcome is the proportion of (guideline-based) prescriptions in relation to the relevant ICD-10 codes as well as daily defined doses and the difference in proportion of certain prescriptions according to guidelines before and after the intervention as compared to the control group. Further outcomes are among others the subjectively perceived risk of antibiotic resistance and the attitude toward the guidelines. The questionnaires to assess this are based on theory of planned behavior (TPB) and health action process approach (HAPA). Variations over time and effects caused by measures other than WASA (Wirksamkeit von Antibiotika-Schulungen in der niedergelassenen Aerzteschaft-Effectiveness of antibiotic management training in the primary health care sector) training are taken into account by including the control group and applying interrupted time series analysis.

**Ethics and Dissemination:** The study protocol and the data protection concept respectively were reviewed and approved by the Ethics Committee of the Hannover Medical School and the Federal Commissioner for Data Protection and Freedom of Information.

**Trial Registration:** https://www.drks.de/drks_web/navigate.do?navigationId=trial.HTML&TRIAL_ID=DRKS00013951, identifier DRKS00013951.

## Introduction

The era of antibiotics began with the discovery of penicillin by Alexander Fleming in 1928 (Fleming, 2001) and it was believed that the fight against infectious diseases was won. However, by the 1940s, the emergence of penicillin resistant strains was evident (Sengupta et al., 2013). Regardless of these early warning signs for the public health threat resistance, the topic of antibiotic stewardship is a fairly recent one (Dyar et al., 2017). Additionally, in spite of its importance, primary health care has not been in focus. In Germany, about 85% of antibiotics in human medicine are prescribed in the outpatient sector and approximately 50% of these by general practitioners (GP) respective approximately 60%, if specialists in internal medicine working as GP in primary health care are included (Bundesamt für Verbraucherschutz und Lebensmittelsicherheit, Paul-Ehrlich-Gesellschaft für Chemotherapie e.V., 2016).

The phenomenon of antibiotic resistance poses a serious threat to global public health as it reduces the effectiveness of treatments for serious bacterial infections and thus increases the risk of fatal outcomes (European Centere for Disease Prevention and Control, 2015; Ventola, 2015). It has been shown that there is an association between the amount of prescriptions and antibiotic resistance (Goossens et al., 2005; Meyer et al., 2013; European Centre for Disease Prevention and Control, 2015; Kim et al., 2018).

Various scientific medical societies systematically develop and release clinical guidelines for the rational prescription of antibiotics for specific diseases. They are based on current scientific findings. In general, guidelines are written by expert groups and either based on consensus or systematic literature reviews. Depending on the methods, they are classified in different development levels from S1 to S3, with S3 being the highest quality level of the development methodology.

Prescribing practice in Germany is often not in line with these guidelines (Fünfstück et al., 2017), even for urinary tract infections (UTI) that are reported to be among the most common bacterial infections among women in the primary health care sector (Dicheva, 2015). For example, broad-spectrum antibiotics such as Amoxicillin or Cefuroxim (axetil) are prescribed despite not being recommended (Bundesamt für Verbraucherschutz und Lebensmittelsicherheit, Paul-Ehrlich-Gesellschaft für Chemotherapie e.V., 2016). In the case of acute respiratory tract infections (ARI), for 90% of episodes, antibiotics are deemed as not indicated (Bundesamt für Verbraucherschutz und Lebensmittelsicherheit, Paul-Ehrlich-Gesellschaft für Chemotherapie e.V., 2016) because a virus being the causative agent. A study monitoring German GP on guideline adherence regarding antibiotic prescriptions for lower respiratory tract infections found that only 52% of the prescriptions were congruent with the guideline (Kraus et al., 2017). In summary, even if Germany’s primary health care sector ranks in the lower third regarding prescriptions compared to other European countries (Bundesamt für Verbraucherschutz und Lebensmittelsicherheit, Paul-Ehrlich-Gesellschaft für Chemotherapie e.V., 2016; European Centre for Disease Prevention and Control, Antimicrobial consumption, 2018) antibiotics are overprescribed and narrow-spectrum antibiotics are chosen too seldomly; hence, antibiotic prescription in primary health care needs improvement.

In Germany, clinical guidelines based on principles of evidence-based medicine are free for download and regularly updated (www.awmf.org). GP seem to value the quality of German guidelines, i.e., less than 10% see a lack of good guidelines; however, less than 40% regularly use them (Salm et al., 2018). The problem might be that the guidelines are extremely long, e.g. for UTI 254 pages and for some conditions, several guidelines with conflicting recommendations exist, making it time-intensive and almost impossible for GP to thoroughly read and incorporate the guidelines into their daily practice; hence, there is a need for concise and targeted trainings for medical doctors working in primary health care.

Several studies with different designs, e.g. focusing on children (de Bie et al., 2016) or online courses (Little et al., 2013) have investigated the effectiveness of training GP on antibiotic prescribing. Due to differences in the health care systems, comparability is not always granted, but most have used either a before-after design without control group or included only a control group, but not both (Roque et al., 2014) or failed to identify the intervention group (Slekovec et al., 2012)–the first one being obsolete because the underlying trend cannot be taken into account (Goodacre, 2015; Bernal et al., 2017), the second one not able to investigate changes by an intervention. There is also a notable lack of studies addressing the impact of UTI antibiotic stewardship training in primary health care (Roque et al., 2014; Drekonja et al., 2015); most studies focused on the hospital setting. Currently, there are several similar projects in different regions of Germany: among them RAI “Rationaler Antibiotikaeinsatz durch Information und Kommunikation,” an interdisciplinary project focusing on Massive Open Online Courses and information material that GP can disseminate to their patients (RAI - Rationaler Antibiotikaeinsatz durch Information und Kommunikation, 2016), and RESIST “RESISTenzvermeidung durch adäquaten Antibiotikaeinsatz bei akuten Atemwegsinfektionen”, a project addressing physicians working in several medical specialties targeting respiratory tract infections (RESIST - Resistenzvermeidung durch adäquaten Antibiotikaeinsatz bei akuten Atemwegsinfektionen, 2020). In both projects the physicians took part in online-training.

Based on these research gaps we developed a study with the following characteristics: interactive classroom trainings with condensed presentation of current clinical guidelines on diagnosis and antibiotic treatment with focus on aspects relevant for primary care and a strong emphasis on respiratory infections while also including UTI.

During these trainings, the desired behavior (prescribing according to the guidelines) has been promoted. WASA is the first study in Lower-Saxony to evaluate the effectiveness of interactive training for GP on antibiotic stewardship.

## Aims and Objectives

WASA “Wirksamkeit von Antibiotika-Schulungen in der niedergelassenen Aerzteschaft” aims to evaluate quantitative and qualitative change in antibiotic prescriptions for acute respiratory and urinary tract infections after attendance of trainings on antibiotic management.

Thus, we want to assess effectiveness of antibiotic stewardship trainings for GP including long-term effects, i.e., over the course of one year. Additional aims are to measure the participants` willingness to attend several trainings, the subjective effectiveness and other sentiments, the experienced quality of the trainings and the overall costs to organize these. The target group are GP in one defined geographical region (model region: South-East-Lower Saxony) all of whom received invitations to attend the trainings.

The main challenge is to link primary and secondary data in a data protection compliant way.

## Materials

For the training sessions, interactive lectures were developed, summarizing the guidelines and including educational case reports for discussions. The participants received printouts of the case reports. All materials were developed for WASA by respective specialists including a review by an independent expert in the field.

To collect primary data, we employed three questionnaires: one to be filled in immediately before the trainings, one afterwards and the third one twelve months later.

The participating GP are supposed to provide some basic information among others on their age category, years of practice and practice size.

Questionnaires for the participating GP were designed based on psychological models which aim to explain and predict (health related) intentions and behavior. Models of behavior change assume there is a pattern of factors that influence motivation and change in behavior. Predictor variables can be identified and it is assumed that the behavior is the outcome of a conscious intention (Schwarzer, 2008).

To give an example, it is assumed that there has to be a positive expectation on the outcome. This is captured by asking “The influence that I myself have on the development of resistance development of resistance, is...” with a 7-Likert scale ranging from “very significant” to “very insignificant”.

The theory of planned behavior (TPB) postulates that there are independent determinants of intention including the so called “subjective norm.” This construct refers to the fact that people feel social pressure regarding the display of a particular behavior (Ajzen, 1991). In concrete, we ask whether the physician feels a) social pressure not to prescribe antibiotics b) thinks that important persons like his or her family, friends colleagues or patients expect them to prescribe antibiotics and c) social pressure to prescribe antibiotics. Answers are to be provided on a 7-Likert-Scale ranging from "completely true" to "not true at all". (Please find the complete questionnaires in the supplement.)

The health action process approach (HAPA) model suggests a distinction between a pre-intentional motivation process and a post-intentional volition process. For instance, it is not sufficient to be aware of a risk; one also has to believe in a high self-efficacy and to anticipate a positive outcome to form an intention. Another important issue in this model is “coping planning” for one has to make several plans regarding how to act if barriers emerge (Schwarzer, 2008).

The questionnaires were designed based on pre-study interviews with nine GP and later pretested with seven all of whom were not eligible for the study.

We used semi-structured interviews for some trainers and participants to assess this from different perspectives.

## Methods

### Design

WASA is a pragmatic (Schwartz and Lellouch, 1967; Roland and Torgerson, 1998) quasi-experimental study which uses a controlled before-after design to compare changes attributable to the interventional trainings while considering general changes due to e.g. overall information on antibiotic stewardship policies by including a control group and applying interrupted time series analysis.

Three separate training sessions on 1) urinary tract infections (UTI), 2) upper (URTI) as well as 3) lower respiratory tract infections (LRTI) were offered and included up-to-date diagnostics and antibiotic prescriptions. Each GP could decide whether he or she was interested in only one, two or all three topics. Participation was voluntary. Taking part in the study was not a requirement to attend the trainings.

WASA partners are among others the regional maximum-care hospital and the “Hygienenetzwerk Südostniedersachsen” (hygiene network South-East-Lower Saxony, HN-SON) which operates in the catchment area of the regional maximum-care hospital since 2009.

### Setting

The study area includes the cities and rural adjunct districts of Brunswick, Gifhorn, Goslar, Helmstedt, Peine, Salzgitter, Wolfenbüttel and Wolfsburg. In this region, there are approximately 750 GP providing primary care who are the target population. In Germany, one needs to be referred by a doctor for any hospital treatment, except for emergencies. In any other case, patients consult a physician working in the primary health care sector during their office hours.

Regional hygiene networks are joint undertakings of health care providers including public health services, in order to combat relevant pathogens through standardized and quality-checked procedures across all medical care facilities. A cooperation of multiple partners provides the opportunity to carry out a project and study like WASA since various expertize and data supply are required.

### Study Population and Recruitment

The study was conducted between 2018 and 2020 in South-East-Lower Saxony, the region the hygiene network operates in (Pfingsten-Würzburg et al., 2011).

As German physicians have to collect 250 Continuing Medical Education (CME) points over five years a number of CME trainings are held in any given German region. There is no coordinated approach as to what topics are covered in what timely matter (Reglement beachten, 2014) but rather it is a self-regulatory process. The WASA trainings were offered within this framework (5 CME points). For our project, all GP from the study region received targeted invitations, letters as well as emails and faxes. Topics and dates of the trainings were also displayed on the webpage of the chamber of physicians. No further in- or exclusion criteria applied.

### Intervention

The voluntary attendance of the trainings is regarded as the intervention. The trainings were designed to be interactive and involved the discussion of educational case reports as well as problems the GP encounter in their professional work regarding the topic. Such educational work is important, as small discussion groups have been shown to be some of the most effective teaching strategies within the framework of antibiotic stewardship (Roque et al., 2014). All lecturers were chosen based on a peer-approach, i.e. active medical doctors from the field, and were trained in a standardized manner and used the same training materials. The 2.25 h trainings focused on condensed guideline recommendations without covering communication skills. No financial compensation for travel, workshop participation or a desired prescribing behavior afterward was rewarded. However, attendees received (independently from participation in the study) free copies of the guidebook “Rationale orale Antibiotikatherapie für Erwachsene im niedergelassenen Bereich” (Rational oral antibiotic therapy for adults in the outpatient sector) by the Governmental Institute of Public Health of Lower Saxony (NLGA), which otherwise costs 10 €. For the attendance of a second or third training participants received something of a similar value as a token of appreciation.

This study is funded by the German Federal Ministry of Health and gets no support from the pharmaceutical industry. The intervention took place in eight different towns within the study region from April 2018 to January 2019. Follow-up lasted until January 2020. The control group consists of all GP from the region that did not attend the trainings.

### Data Collection and Processing

Data were collected on three levels: 1) we employed the above-mentioned questionnaires, 2) we use health records of the region provided by AOK Niedersachsen and 3) some of the trainers and participants were interviewed right after the trainings with the aim to evaluate the acceptance of this (standardized) training format. The latter represents the qualitative part of the analysis.

In Germany, about 90% of the population were insured with a statutory health insurance company (Die gesetzlichen Krankenkassen, 2021); in 2017 out of these, about 36% with the AOK–Die Gesundheitskasse (AOK) (Daten des Gesundheitswesens, 2017). This translates to approximately 2.7 million individuals in Lower-Saxony (AOK–Die Gesundheitskasse für Niedersachsen Geschäftsbericht, 2017). Statutory health insurances companies collect data on a quarterly basis for each doctor and practice regarding the date of birth, sex, diagnoses (ICD-10 codes) whereas prescriptions (ATC (Anatomical Therapeutic Chemical)-codes) of each patient are recorded on a daily basis; primarily for billing reasons. In WASA, the antibiotic prescriptions will be related to the ICD-10 codes in 3-months-intervals and compared to the recommendations from the guidelines. To do this, the training material based on the guidelines had to be screened for diagnoses and the corresponding ICD-10 code (www.icd-code.de/) has to be looked up. Similarly, the ATC classifications for the recommended antibiotics have to be looked up (www.whocc.no/atc_ddd_index/). Based on the recommendations in our training material, we linked both. For further details, please see Table 1.

**TABLE 1:** Antibiotics and corresponding indications.

Antibiotic	ATC	Indications (ICD-10) UTI	Indications (ICD-10) URI	Indications (ICD-10) LRI	AWaRe Classification Database
Doxycycline	J01AA02	x	J01.-, J06.8, J06.9, H66.0, H66.4, H66.9	J13, J14, J15.-, J16.-, J18.-, J44.0-, J44.1	access
Amoxicillin	J01CA04	x	J01.-, J06.8, J06.9, H66.0, H66.4, H66.9	J13, J14, J15.-, J16.-, J18.-, J44.0, J44.1	access
Pivmecillinam	J01CA08	N30.0, N30.3, N30.8, N30.9, N39.0, N39.88, N39.9 O23.- (excl.: O23.0, O23.5)	x	x	access
Phenoxymethylpenicillin	J01CE02	x	J02.-, J03.-, J06.-	x	access
Sultamicillin	J01CR04	x	J32.-	x	access
Amoxicillin-clavulananic acid	J01CR22	N10, N12	J01.-, J06.8, J06.9, J32.-, H66.0, H66.4, H66.9	J13, J14, J15.-, J16.-, J18.-	access^a^
First-generation cephalosporins^b^	J01DB	x	J02.-, J03.-, J06.-	x	access
Cefalexin^c^	J01DB01	x	J02.-, J03.-, J06.-	x	access
Cefuroxim	J01DC02	N30.0, N30.3, N30.8, N30.9, N39.0, N39.88, N39.9 O23.- (excl.: O23.0, O23.5)	x	x	watch
Cefpodoxime (proxetil)	J01DD13	N10, N12, N30.0, N30.3, N30.8, N30.9, N39.0, N39.88, N39.9, O23.- (excl.: O23.5), T83.5, T83.8, T83.9	J01.-, J06.8, J06.9, H66.0, H66.4, H66.9	x	watch
Trimethoprim and derivates	J01EA	N10, N12, N30.0, N30.1 N30.2, N30.3, N30.8, N30.9, N39.0, N39.88, N39.9	x	x	access^d^
Sulfamethoxazole and Trimethoprim (Cotrimoxazol)	J01EE01	N10, N12, N30.0, N30.1 N30.2, N30.3, N30.8, N30.9 N39.0, N39.88, N39.9	x	A37.-, J17.0	access
Macrolides^b^	J01FA	x	J02.-, J03.-, J06.-	x	watch
Clarithromycin^c^	J01FA09	x	x	A37.-, A48.1, J13, J14, J15.-, J16.-, J17.0, J18.-, J44.0-, J44.1-	watch
Azithromycin	J01FA10	x	x	A37.-, J17.0	watch
Clindamycin	J01FF01	x	J02.-, J03.-, J06.-, J32.-	x	access
Ciprofloxacin	J01MA02	N10, N12, N30.0, N30.1, N30.2, N30.3, N30.8, N30.9, N39.0, N39.88, N39.9, T83.5, T83.8, T83.9	x	x	watch
Levofloxacin	J01MA12	N10, N12, N30.0, N30.3, N30.8, N30.9, N39.0, N39.88, N39.9, T83.5, T83.8, T83.9	J01.-, J06.8, J06.9, H66.0, H66.4, H66.9	J13, J14, J15.-, J16.-, J18.-	watch
Moxifloxacin	J01MA14	x	J32.-	J13, J14, J15.-, J16.-, J18.-	watch
Nitrofurantoin	J01XE01	N30.0, N30.1, N30.2 N30.3, N30.8, N30.9, N39.0, N39.88, N39.9	x	x	access
Fosfomycin	J01XX01	N30.0, N30.1, N30.2,N30.3, N30.8, N30.9, N39.0, N39.88, N39.9 O23.- (excl: O23.0, O23.5)	x	x	watch
Nitroxoline	J01XX07	N30.0, N30.3, N30.8, N30.9, N39.0, N39.88, N39.9	x	x	x

The table is based on the official ATC index 2017 available at dimdi.de and sorted accordingly. “x” denotes that the antibiotic is not indicated for this group of diagnoses.

^a^ ATC code J01CR22 existed 2016–2018.

^b^ Whole class of antibiotics correct for the listed indications according to the training material.

^c^ Belonging to a class of antibiotics but specifically mentioned in the training material.

^d^ Classification “Access” only valid for J01EA01.

Statutory health insurance routine data will be available for a period of two years before and one year after the intervention.

After the completion of the training phase, non-response questionnaires were send out to all GP of the region to gather some basic information that cannot be obtained from the statutory health insurance routine data. Due to the reason that we do not know the names of the participants, we asked those who participated to ignore the letter.

Data collection will be completed by the end of 2020.

**FIGURE 1:**
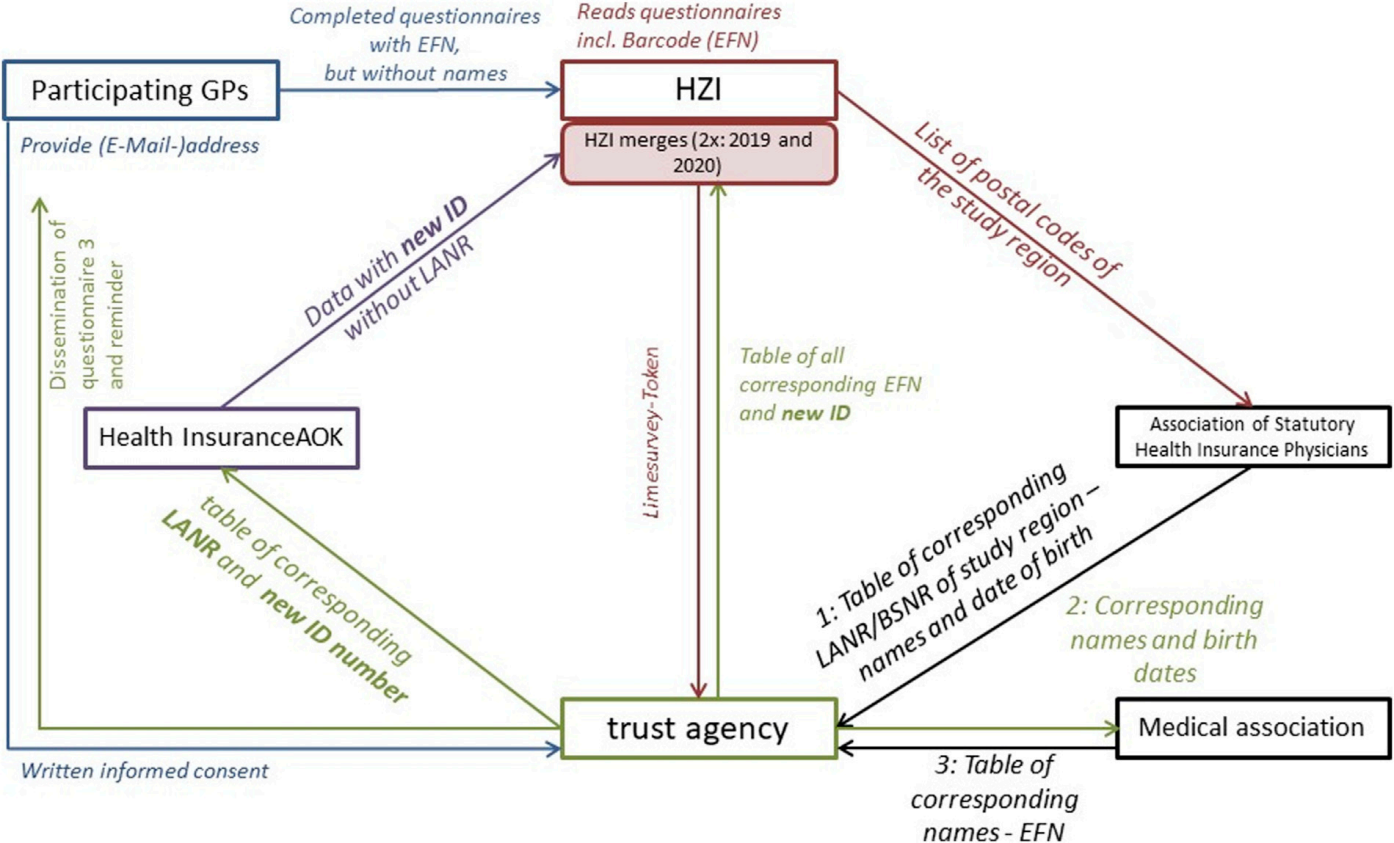
Data Flow-matching of primary and secondary data with respect to data protection requirements. HZI: Helmholtz-Zentrum für Infektionsforschung, GP: general practitioners, EFN: number used to collect CME points, LANR: personal physician number, BSNR: practice number.

For the data analysis, it is important to connect the primary data from the questionnaires with the secondary data provided by the statutory health insurance. However, no institution involved in the project except the trust agency should be aware of who attends the trainings, participates in the study, or the amount of specific antibiotic prescriptions. Therefore, we developed a data flow that works on the prerequisite that data which identify any participant to be kept separated from questionnaire and statutory health insurance routine data (see Figure 1). For this unique data flow we made use of three numbers each physician (practicing in the primary health care sector) is assigned by the German health system: the practice number (“Betriebsstättennummer,” BSNR), the personal physician number (“Lebenslange Arztnummer,” LANR) and the number used to collect CME points (“Einheitliche Fortbildungsnummer,” EFN). The latter is used to provide identification to connect the 3 questionnaires. It consists of 15 digits of which six are generated randomly and does not allow identification of any doctor with the exception of the Chamber of Physicians (“Ärztekammer”) which provides the number (pseudonym).

A trust agency has been employed to match the EFN to the LANR and BSNR. For this purpose, the agency received lists from the association of Statutory Health Insurance Physicians (“Kassenärztliche Vereinigung”) and the Chamber of Physicians with the names and years of birth of all GP in the region so LANR/BSNR can be assigned to the respective EFN.

New study IDs for all GP of the study region have been created by the trust agency and assigned to the EFNs and respective LANRs and BSNRs. A list was given to the AOK so that their data on diagnosis and antibiotics prescribed could be provided with new ID that do not allow others to draw conclusions about the respective person. In a second step, a list that matches the new ID to the EFN was passed to the researchers at Helmholtz Centre for Infection Research (HZI). Here, all EFN that correspond to an EFN on any questionnaire are identified to belong to GP of the intervention group; those GP that did not fill in questionnaires, i.e. did not provide EFN, are assigned to the control group. Physicians who participated in the trainings but not in the study were asked to provide their EFN so that they can be excluded from the control group. The AOK passes on all routine data of all the GP from the study region covering the years 2016 till the beginning of 2020, without distinguishing between participants and non-participants. Based on the new ID, these data can be matched to the questionnaire data as displayed in Figure 1.

In some of the guidelines, the corresponding ICD-10 is listed, but not in all and neither in the book “Rationale orale Antibiotikatherapie für Erwachsene im niedergelassenen Bereich” (Rational oral antibiotic therapy for adults in the outpatient sector) nor in the training materials directly based on the guidelines. ATC-codes are not of interest to the GP and therefore not mentioned in the guidelines or training materials but are found in the health insurance data whereas there the name(s) of the recommended antibiotic(s) is/are lacking. Hence, we collected all of this information from the training materials and the book and complemented with information from the guidelines in one table (Table 1) which forms the basis for the analyses of the proportion of (guideline-based) prescriptions in relation to the relevant ICD-10 codes as well as daily defined doses and the difference in proportion of certain prescriptions according to guidelines before and after the intervention as compared to the control group. Additionally, this table includes a column which is based on the WHO AWaRe Classification Database which contains details of 180 antibiotics classified as access, watch or reserve (Essential medicines and health products-WHO releases the 2019 AWaRe Classification Antibiotics, 2019).

### Outcomes

The primary outcome is the effectiveness of the antibiotic management trainings including long-term effects over one year, i.e. the quantitative and qualitative change in antibiotic prescriptions for acute respiratory and urinary tract infections derived from the statutory health insurance routine data. Concrete: the proportion of (guideline-based) prescriptions in relation to the corresponding ICD-10 codes, e.g. N30.0 as well as the overall amount of prescribed antibiotics commonly prescribed for the diseases of interest expressed in daily defined doses and the change over time. To asses this, the ratio of recommended antibiotics listed in Table 1 based on the training materials and the German guidelines and therefore considered as correct prescriptions as opposed to all other antibiotics (the remaining in J01) will be calculated. The quality indicators will be build based on prescriptions for the diseases of interest in relation to all diagnoses of interest and compared to the recommended percentage of proposed disease specific antibiotic prescribing quality indicators in Europe which are 0–30% for bronchitis/bronchiolitis, 0–20% for acute siniusitis whereas for female patients with urinary tract infection it is 80–100% and for pneumonia 90–100% for all patients (Adriaenssens et al., 2011).

Other measures are costs and number of attended trainings per GP, the GP’s subjectively perceived gain of knowledge and the change in the perceived extent of the own influence on the development of antibiotic resistance and the determination of the intention of the GP to change or maintain their prescribing behavior.

Questionnaires deployed in WASA are mainly based on HAPA and theory of planned behavior (“subjective norms”) models. This allows us e.g. to report on the perceived change in prescription behavior.

### Analyses

To analyze the effectiveness of the trainings, an interrupted time series regression will be applied. This method is increasingly being used to evaluate the effect of different public health interventions (Bernal et al., 2017; Hategeka et al., 2020) as in (Santa-Ana-Tellez et al., 2013; Spoelman et al., 2016). It requires some a priori hypotheses on the impact of the intervention on the outcome (Bernal et al., 2017). Antibiotic prescriptions seem to decrease in Germany–especially for children–but to be stable among GP. Studies have shown that prescriptions which are not in line with the guidelines can be reduced by 10 to 20% after having attended trainings on this topic (Schulz et al., 2014; Kraus et al., 2017).

The analyses will be conducted for all topics together and also for each topic separately. We will compare participants’ and non-participants’ patients regarding patient’s age and sex. If there are major significant differences, we will include the corresponding variable in the interrupted time series analysis to control for it.

### Ethics, Data Protection and Dissemination

The study protocol was reviewed and approved by the ethics committee of Hanover Medical School and the data protection concept was reviewed and approved by the Federal Commissioner for Data Protection and Freedom of Information (“Bundesbeauftragte für den Datenschutz und die Informationsfreiheit,” BfDI).

## Discussion

Despite low antibiotic consumption in Germany in comparison to other European countries, improvement of antibiotic prescription in primary health care is necessary in Germany, e.g. by less prescribing of broad spectrum antibiotics as is recommended in the guidelines. We conduct a study on the effectiveness of interactive trainings for GP regarding UTI and ARI in a model region. If the trainings prove to be successful, findings from this study can be used for further projects.

To our knowledge, the design of our study is a unique selling point. It has been proven that data protection conform linkage of questionnaire data and health insurance data is possible by applying our concept. Our study has several strengths and limitations. Data are collected on various levels. Evaluation involves quantitative (questionnaires and statutory health insurance routine data) and qualitative aspects in order to cover various aspects and to provide a full picture. Our concept allows connection of primary and secondary data whilst ensuring personal data protection.

However, by attending the trainings the GP assigned themselves to the intervention group which might have resulted in a self-selection bias. Due to the abundance of professional trainings for GP with several topics it can be assumed that those GP who attended the trainings on antibiotic stewardship are more aware of the problem of resistance and more eager to stay up to date than those who stay away and their prescribing behavior might differ independent of our intervention.

One advantage of health insurance data clearly is that many patients can be included without time-consuming data collection. Health insurance data are not subject to recall bias. However, they only provide data on age and sex of the patients as well as diagnoses and medication but no additional data. Another disadvantage is that prescriptions are available on a daily basis whereas diagnoses are only available on a quarterly basis. Hence, prescriptions cannot be linked directly to diagnoses. This does not pose a big problem if there are very few diagnoses and prescriptions for one specific patient. Also, if e.g. Nitrofurantoin has been prescribed; this is recommended for urinary tract infections but not for respiratory tract infections, that is why assignment is relatively clear. But if this is a patient with multiple diagnoses and prescriptions it becomes complicated since antibiotics like Levofloxacin are recommended to cure urinary tract infections, upper respiratory tract infections and lower respiratory tract infections (and for further infections which have not been addressed in our trainings but of course can occur among the GP’s patients) This can be approached by conducting sensitivity analyses and excluding all patients with more than one diagnoses of interest from the analyses.

We will bring our results into context with other published literature in this field. If the trainings prove to be effective, recommendations for future concepts of antibiotic stewardship training for primary health care can be given.

As opposed to other studies, WASA does not offer any financial benefit for the participants which could be a disadvantage during the recruitment process but on the other hand financial incentives are not necessarily sustainable and long-term effective.

## Data Availability

The original contributions presented in the study are included in the article/Supplementary Material, further inquiries can be directed to the corresponding authors.
