# Supplementary material for: Effectiveness of Trainings of General Practitioners on Antibiotic Stewardship: Methods of a Pragmatic Quasi-Experimental Study in a Controlled Before-After Design in South-East-Lower Saxony, Germany (WASA)
Source: Front Pharmacol. 2021 Apr 22;12:533248. doi: 10.3389/fphar.2021.533248 (PMC8103612; doi:10.3389/fphar.2021.533248)
Supplement: Supplementary file 1 [file datasheet1.docx]

**WASA questionnaire no. 1**

Dear participant,

We are pleased that you are participating in our training. We would like to know how you like the training and, with your help, find out where there is a need for optimization.

We are further interested in the impact of our events, i.e., whether they bring about a change in your intentions with regard to your prescribing practice.

For this reason, we ask you to fill in questionnaires before and right after the trainings. This is always on a voluntary basis. You have the option of leaving individual questions unanswered.

All your information will be treated confidentially and will not be linked to your name.

Therefore, please do not write your name at any point on the questionnaires. Please do not write your name on the questionnaires, just enter your EFN (number used to collect CME points) or affix it on the field provided for this purpose.

We thank you very much and wish you a pleasant seminar!

The WASA study team

Question 1: Which statement do you agree with in the context of respiratory or urinary tract infections?
*Scale: 1 - completely true - to 7 - not true at all*

- I advise my patients to take phytopharmaceuticals instead of antibiotics.
- I advise my patients to take homeopathic medicines instead of antibiotics.
- I advise my patients to take phytopharmaceuticals in addition to antibiotics.
- I advise my patients to take homeopathic medicines in addition to antibiotics.

Question 2: Have you recently prescribed antibiotics to patients with symptoms of urinary or respiratory tract infections in accordance with the guidelines of the medical societies? Please tick the one answer that most applies to you.

- Yes, and the guideline-compliant prescription for these indications has become routine for me.
- Yes, but the guideline-compliant prescription for these indications has not yet become routine for me.
- No, but I have the firm intention to make it my routine.
- No, but I am thinking about routinely prescribing antibiotics for these indications according to guidelines.
- No, and I am not planning to routinely prescribe antibiotics for these indications according to guidelines.

Question 3: Please imagine the following situation: A patient comes to your practice with symptoms of an upper or lower respiratory tract infection or a urinary tract infection. What applies?

*Scale: 1 - completely true - to 7 - not true at all*

- I feel social pressure not to prescribe an antibiotic.
- Most of the people who are important to me (family, friends, colleagues, patients) expect me to prescribe an antibiotic.
- I feel social pressure to prescribe an antibiotic.

Question 4: What opinion would people or groups likely have if you were to prescribe an antibiotic in the situation described in question 3?

*Scale: 1 - completely true - to 7 - not true at all*

- My patients would approve it for urinary tract infections ^[[1]](#footnote-1)^.
- I myself would approve it for urinary tract infections.
- My practice staff would approve it for urinary tract infections.

Question 5: What do you think: In the situation described at the beginning, what influences colleagues’ prescribing practices of antibiotics for respiratory or urinary tract infections? *Scale: 1 - completely true - to 7 - not true at all*

- Patients' desire for an antibiotic
- Uncertainty and fear of consequences of non-prescribing
- The assumed advantage of the rapid effectiveness of antibiotics
- They have always done it this way

Question 6: How important is the opinion of the following groups of people to you?
*Scale: 1 - completely true - to 7 - not true at all*

- I care what patients think about me.
- I care what my medical colleagues think about me.
- I care what my practice staff thinks about me.

Question 7: Which statements are true in your opinion?
*Scale: 1 - completely true - to 7 - not true at all*

- The guidelines provide evidence-based recommendations for choosing the right antibiotic.
- The guidelines are only an aid to decision-making in treatment.

Question 8: What reasons motivated you to participate in a workshop on the topic of "guideline-compliant antibiotic management"?
*Scale: 1 - completely true - to 7 - not true at all*

- I want to gain knowledge in a short time.
- I want to maintain contact with colleagues and exchange ideas with them.
- I am particularly interested in this topic.
- I receive CME points.
- Others, namely: _____

Question 9: In which situations do colleagues prescribe antibiotics for any of: upper respiratory infections, lower respiratory tract infections, or urinary tract infections without a clear medical indication? *Scale: 1 - completely true - to 7 - not true at all*

- When patients vehemently demand an antibiotic despite good advice.
- When colleagues want to avoid complications from the infection at all costs.
- When the weekend or holidays are just around the corner.
- When the patient is very persistent and comes to the practice several times.

Question 10: Which statement do you think is true?
*Scale: 1 – very significant - to 7 – very insignificant*

- The influence that I myself have on the development of antibiotic resistance is...
- The development of antibiotic resistance for public health is...
- Infections not treatable with antibiotics are… to the public health

Question 11: In accordance with the guidelines only, what effects do you expect if, after appropriate diagnostics, you prescribe antibiotics for the three types of infection mentioned above - urinary tract infections, upper respiratory tract infections, and lower respiratory tract infections
*Scale: 1 - completely true - to 7 - not true at all*

- My patients then get better more quickly.
- I then contribute to minimizing the development of antibiotic resistance.
- My patients are then (more) satisfied with my treatment.
- Many patients then come to my practice with a deterioration and still need an antibiotic.

Question 12:

- My specific goal with regard to my antibiotic prescribing practice is: ____

Question 13: How do you rate yourself?
*Scale: 1 - significantly more - to 7 - significantly less*

- In terms of antibiotics: I prescribe in relation to my specialist group....

Finally, we would like to ask you to provide some demographic data and further information.

Question 14: Your age in years:

- <= 40
- 41 – 65
- > 65

Question 15: Your sex:

- Female
- Male
- Other

Question 16: How many cumulative years have you practiced medicine so far? Please subtract e.g. parental leave or other periods without medical practice.

- < 5
- 5 – 15
- 16 – 25
- 26 – 35
- > 35

Question 17: Which practice option applies to you?

- Individual practice
- Joint practice
- Collaborative practice
- I am employed in a practice.
- Medical on-call service
- Continuing medical education
- Other, namely: _____

Question 18: How many patients come to your practice per quarter on average?

- < 500
- 501 – 1000
- 1001 – 1500
- 1501 – 2000
- > 2000

Questions 19: How did you become aware of the training?

- Internet
- Letter HZI/Medical Association
- Flyer
- Medical Journal
- Colleagues
- Through the General Practitioners' Association
- Others, namely: _____

Question 20: What was the travel duration for attendance at the training?

- < 20 mins
- 20 – 40 min
- 41 – 60 min
- > 60 min

Question 21: Before the training, were you aware of the evaluation of the training courses by the Helmholtz Centre for Infection Research (WASA)?

- Yes
- No

Question 22: Have you participated in any training with a similar topic (online if applicable) in the past 18 months?

- Yes
- No

Have you participated in the online training courses of RESIST^[[2]](#footnote-2)^?

- Yes
- No

Thank you for completing the questionnaire!

Your WASA study team

Enjoy the seminar!

**WASA questionnaire no. 2**

Dear participant,

Thank you very much for participating in our training seminar. Finally, we would like to ask you to answer six short questions.

You have already answered some of the questions in the same or similar form before the training seminar began. By answering the questions, you will help us to assess the quality and effectiveness of the training.

Thank you

The WASA study team

Question 1: Which statement applies?
*Scale: 1 – very significant - to 7 – very insignificant*

- The influence that I myself have on the development of resistance development of resistance is...
- The development of resistance for public health is...
- Infections not treatable with antibiotics are… to the public health

Question 2: When you think about your everyday practice, in which situations would you find it easy to refrain from writing an antibiotic prescription for respiratory or urinary tract infections, unless it is indicated? *Scale: 1 - completely true - to 7 - not true at all*

- When I am sure it is not a bacterial infection.
- If the patients do not wish to receive antibiotics by their own decision.
- When I have enough time to explain to the patients that antibiotics would not have any effect.
- If I had educational materials available.
- If I had a good rapid test (point-of-care test) available.

Question 3: Imagine a patient with a respiratory infection who, despite of you informing her or him, insistently demands the prescription of an antibiotic that is not medically indicated. How do you deal with this?
*Scale: 1 - completely true - to 7 - not true at all*

- I accept the patient's wish and prescribe an antibiotic.
- I suggest a follow-up visit in a few days and do not prescribe anything yet.
- I recommend to the patient to go to another practice in the future.

Question 4: In the long term, what would help you to maintain the guideline-compliant prescription of antibiotics for the three types of infections discussed here - urinary tract infections, upper respiratory tract infections and lower respiratory tract infections?
*Scale: 1 - completely true - to 7 - not true at all*

- Better education of the general population
- A classification of the prescription figures for antibiotics by the Association of Statutory Health Insurance Physicians in comparison to other practices as feedback to me
- Colleagues who also prescribe antibiotics according to guidelines
- Reference books for my practice
- A good training for proper communication with patients

Question 5: How confident are you that you can implement what you have learned in this training in your everyday practice? *Scale: 1 - completely true - to 7 - not true at all*

- I am very confident that I will be able to implement what I have learned in everyday practice.
- Possible reasons for this: ____

Question 6: Evaluation of the training

In order to better assess and further improve the events, we would like to ask you to answer the following questions. Your honest information is extremely important to us. *Scale: 1 - completely true - to 7 - not true at all*

- I rate my own knowledge gain as high.
- The course was adequately prepared and structured.
- The spatial equipment was adequate.
- The duration of the training was appropriate.
- The contents of the training were presented appropriately.
- The contents could be dealt with appropriately in the time.
- The workshop was appropriately interactive.
- There were stimulating discussions among the participants.

We wish you a safe journey home!

**WASA questionnaire no. 3**

Dear Participant,

A few months ago you attended one or more of our WASA trainings and kindly agreed to be invited to fill in another and final questionnaire.
In doing so, you are making a valuable contribution to the scientific evaluation of the training seminars.
If you have any questions, please do not hesitate to contact the Trust Office at WASA.Treuhandstelle@mh-hannover.de.

Thank you very much for your cooperation.

The WASA study team

Your TOKEN (key to be able to assign this questionnaire pseudonymously): *(8-digit alpha-numerical TOKEN)*

Date: __:__:__

Question 1: What has helped you to maintain the guideline-compliant prescribing of antibiotics for the three types of infections discussed here - urinary tract infections, upper respiratory tract infections and lower respiratory tract infections - in the long term?

*Scale: 1 - completely true - to 7 - not true at all*

- Better education of the general population
- A classification of the prescription figures for antibiotics by the Association of
- Statutory Health Insurance Physicians in comparison to other practices as feedback to me
- Colleagues who also prescribe antibiotics according to guidelines
- Reference books for my practice
- A good training for proper communication with patients
- Something else helped me with this, and that is: ____

Question2: How confident are you that after an episode of non-guideline prescribing you will be able to implement again what you have learned in the seminar?
*Scale: 1 - completely true - to 7 - not true at all*

- Such episodes do not occur.

Question 3: Have you recently prescribed antibiotics to patients with symptoms of

antibiotics in accordance with the guidelines of the medical societies?

- Yes, and the guideline-compliant prescription for these indications has become routine for me.
- Yes, but the guideline-compliant prescription for these indications has indications has not yet become routine for me.
- No, but I have the firm intention to make it my routine.
- No, but I am thinking about routinely prescribing antibiotics for these indications according to guidelines.
- No, and I am not planning to routinely prescribe antibiotics for these indications according to guidelines.

Question 4: How much of what you learned in the seminar(s) you attended can you implement in your everyday practice?

| 0% | ca. 25% | ca.50% | ca. 75% | 100% |
| --- | --- | --- | --- | --- |

- Possible reasons for this: ____

Question 5: Which statements are true in your opinion? *Scale: 1 - completely true - to 7 - not true at all*

- The guidelines provide evidence-based recommendations for choosing the right antibiotic.
- The guidelines are only an aid to decision-making in treatment.

Question 6: In which situations do colleagues prescribe antibiotics for upper or lower respiratory tract infections or urinary tract infections without a clear medical indication? *Scale: 1 - completely true - to 7 - not true at all*

- When patients vehemently demand an antibiotic despite good advice.
- When colleagues want to avoid complications from the infection at all costs.
- When the weekend or holidays are just around the corner.
- When the patient is very persistent and comes to the practice several times.

Question 7: Which statement do you think is true?
*Scale: 1 – very significant - to 7 – very insignificant*

- The influence that I myself have on the development of resistance development of resistance is...
- The development of resistance for public health is...
- Infections not treatable with antibiotics are… to the public health

Question 8: Imagine a patient with a respiratory infection^[[3]](#footnote-3)^ who, despite of you informing her or him, massively demands the prescription of an antibiotic that is not medically indicated. How do you deal with this? *Scale: 1 - completely true - to 7 - not true at all*

- I accept the patient's wish and prescribe an antibiotic.
- I suggest a follow-up visit in a few days and do not prescribe anything yet.
- I recommend to the patient to go to another practice in the future.

Question 9: In the last twelve months, what difficulties have you encountered when prescribing antibiotics according to guidelines? *Scale: 1 - completely true - to 7 - not true at all*

- I am met with a lack of understanding on the part of the patients.
- Fewer patients come to my practice.
- I encounter resistance from the practice staff because the staff have to deal with patient dissatisfaction.
- Others, namely: _____

We thank you for participating in the study!

1. Urinary, upper or lower respiratory tract infection, depending on which training was held [↑](#footnote-ref-1)
2. Study with a similar topic but different design [↑](#footnote-ref-2)
3. Urinary, upper or lower respiratory tract infection, depending on which training was held [↑](#footnote-ref-3)
